# Supplementary material for: Melting temperatures of MgO under high pressure by micro-texture analysis
Source: Nat Commun. 2017 Jun 5;8:15735. doi: 10.1038/ncomms15735 (PMC5465366; doi:10.1038/ncomms15735)
Supplement: Supplementary Information — Supplementary Figures, Supplementary Tables, Supplementary Notes and Supplementary References. [file ncomms15735-s1.pdf]

Supplementary information

Supplementary Figures

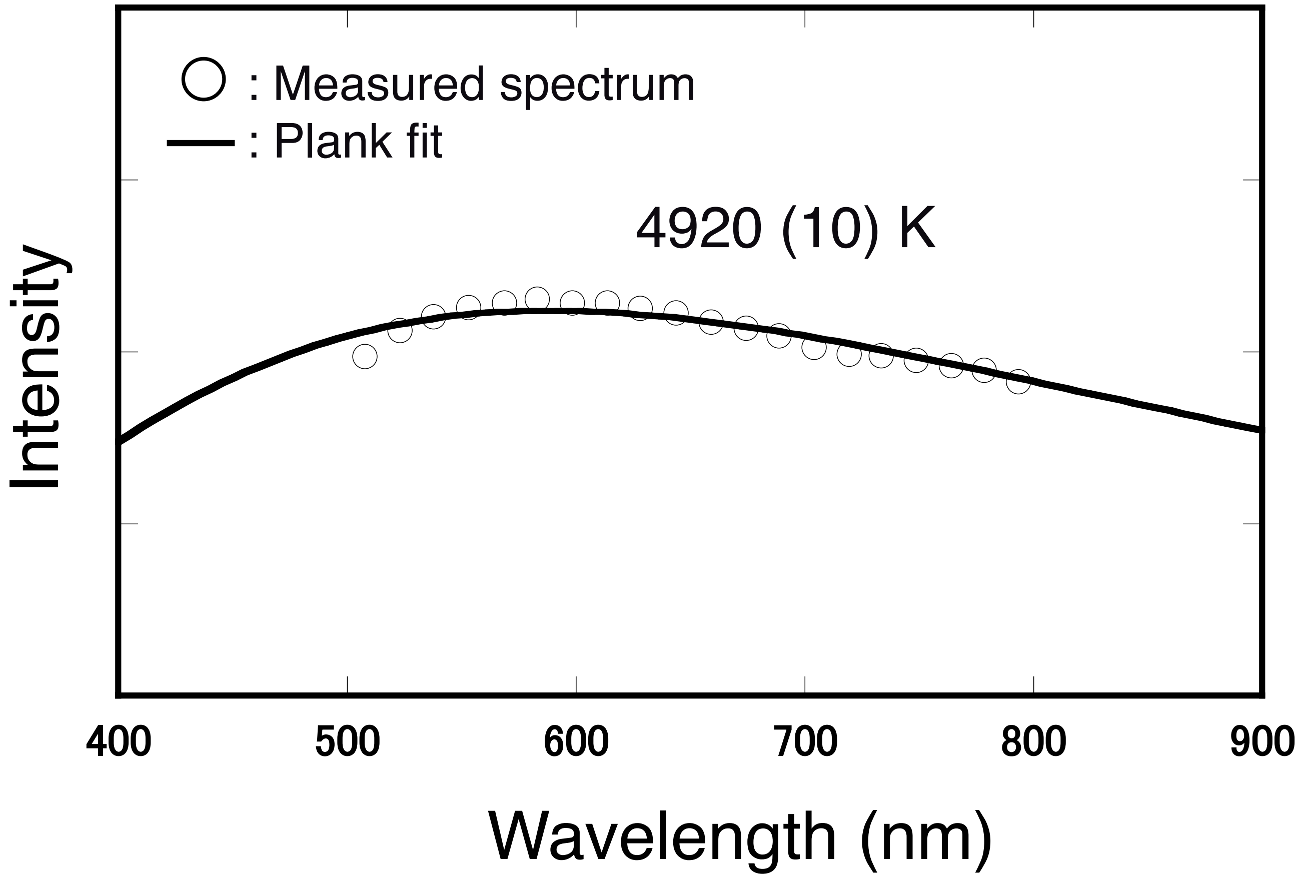

Supplementary Figure 1. Typical example of the thermal emission spectrum of MgO under laser-heating at 18 GPa. The circles and curve represent the radiation data and the fitting by the Planck formula, respectively. The temperature of the present spectrum was determined to be 4920 (10) K.

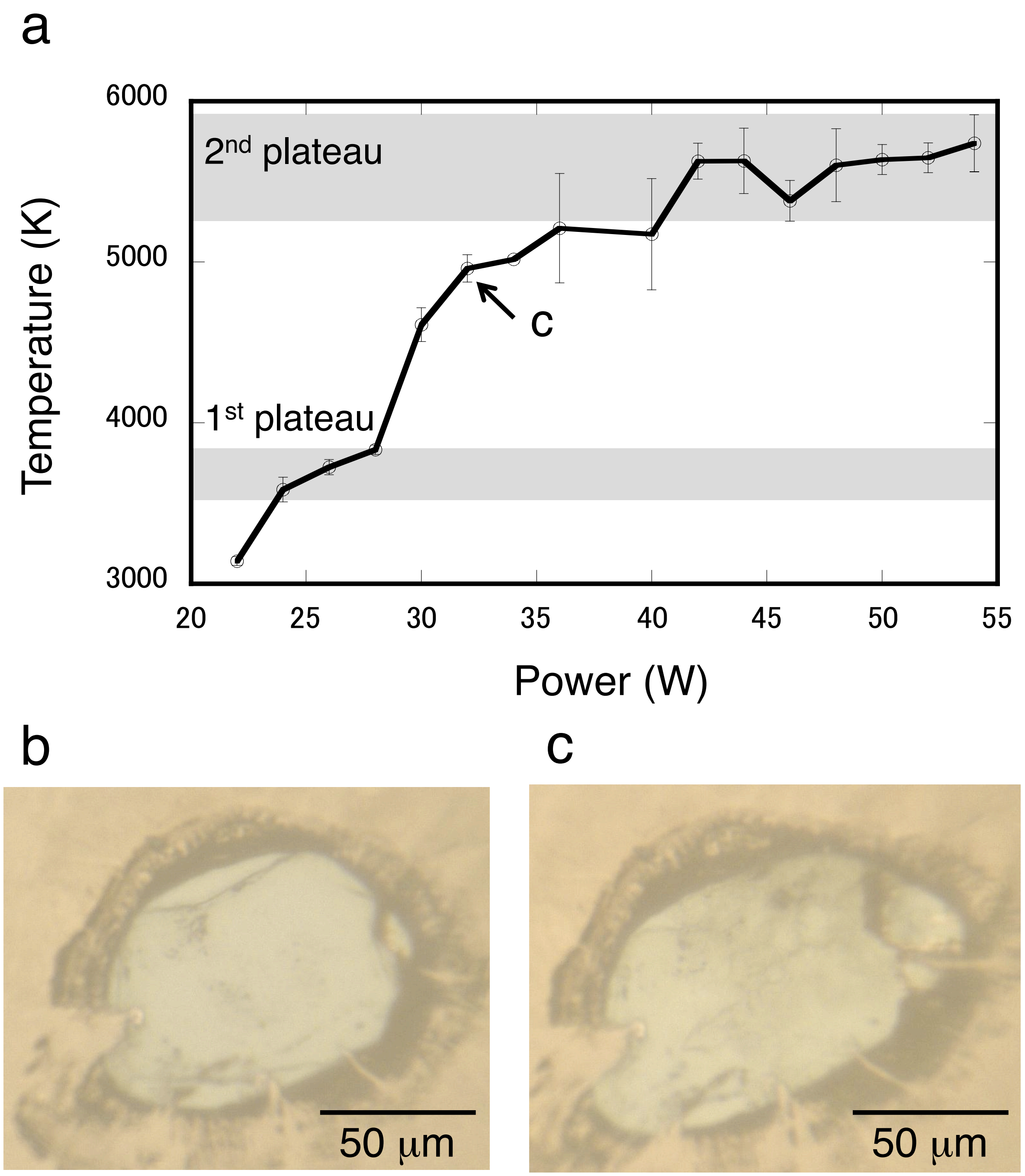

Supplementary Figure 2. Relationship between temperature and the laser power in laser-heating at 46 GPa (a) and microscopic images of the sample before (b) and after heating up to ~5000 K (c). The sample was found to have expanded by ~20 % in the lateral direction after quenching from ~5000 K.

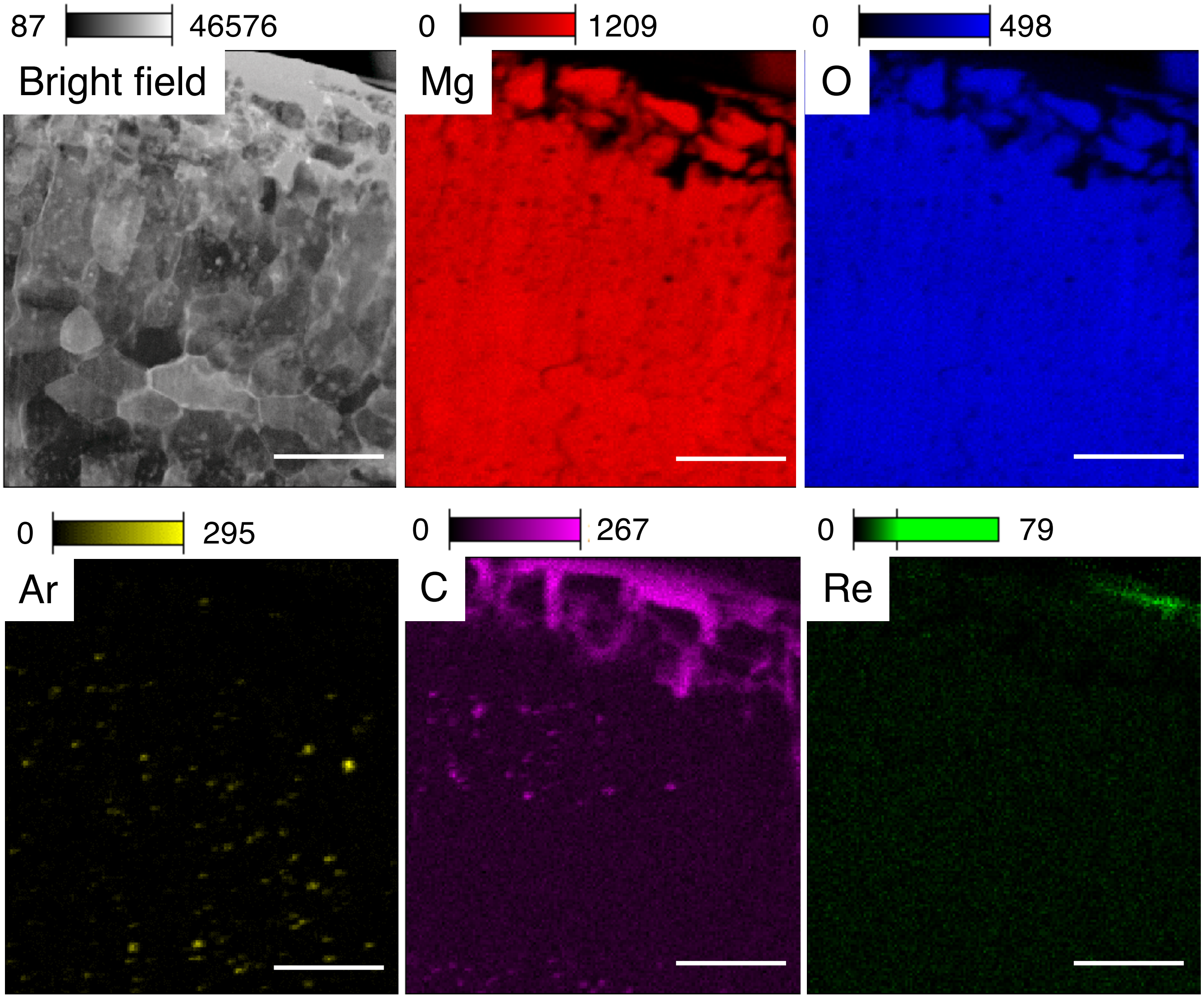

Supplementary Figure 3. Bright-field scanning TEM image and X-ray maps of Mg, O, Ar, C, and Re collected from the laser-heated area of the sample recovered from 32 GPa and 5200 K. Scale bar represents 2 μm. The X-ray maps identified that the quenched sample consists of elongated and granular MgO crystals with many small Ar inclusions. The concentrations of C and Re are artifacts, derived from the epoxy resin and re-deposition from the sputtered Re gasket during Ar ion milling, respectively.

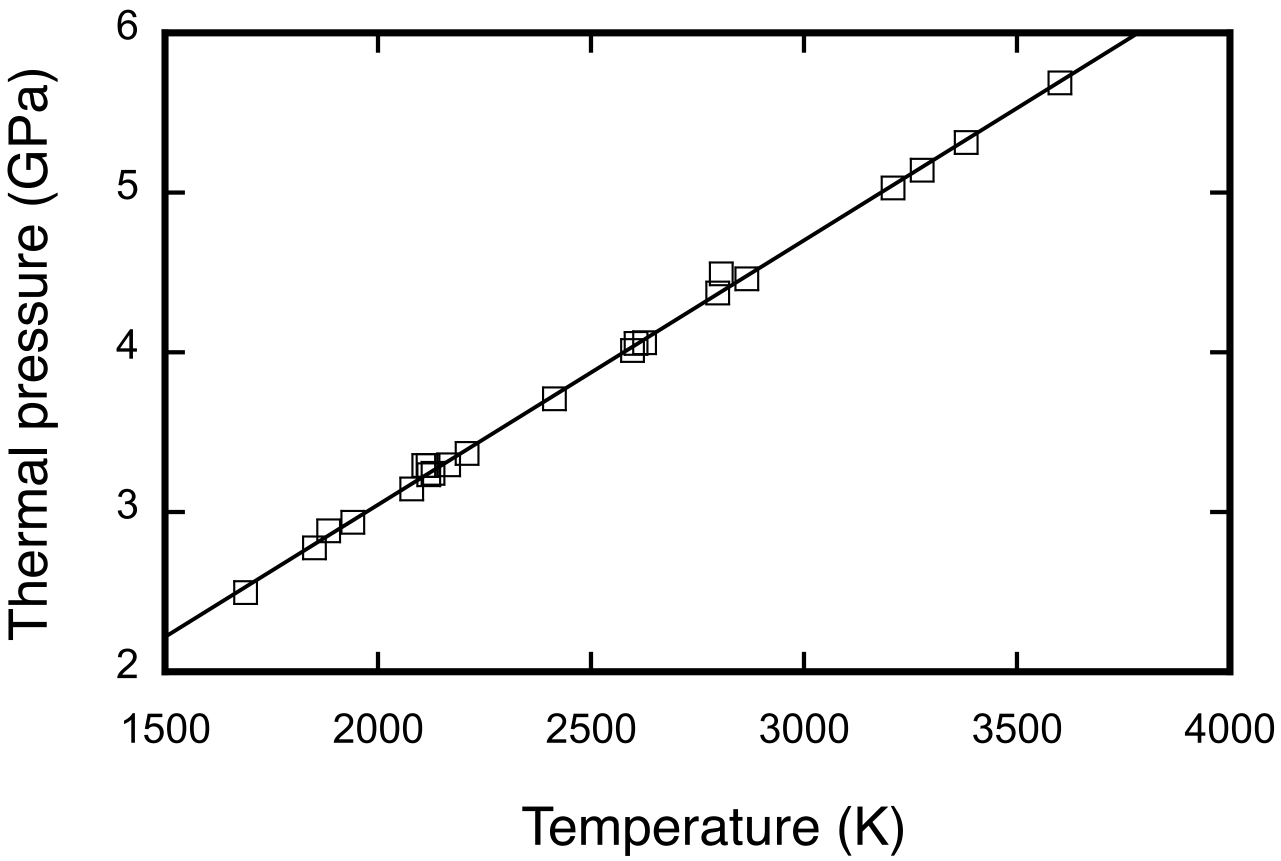

Supplementary Figure 4. Thermal pressure (P_{th}) of MgO under heating as a function of temperature. The squares represent P_{th}s of the thermal EOS data obtained by static compression experiments using a CO_2 laser [1], which were estimated based on the Mie-Gruneisen-Debye model. The linear fit to the P_{th}s is also shown as the solid line.

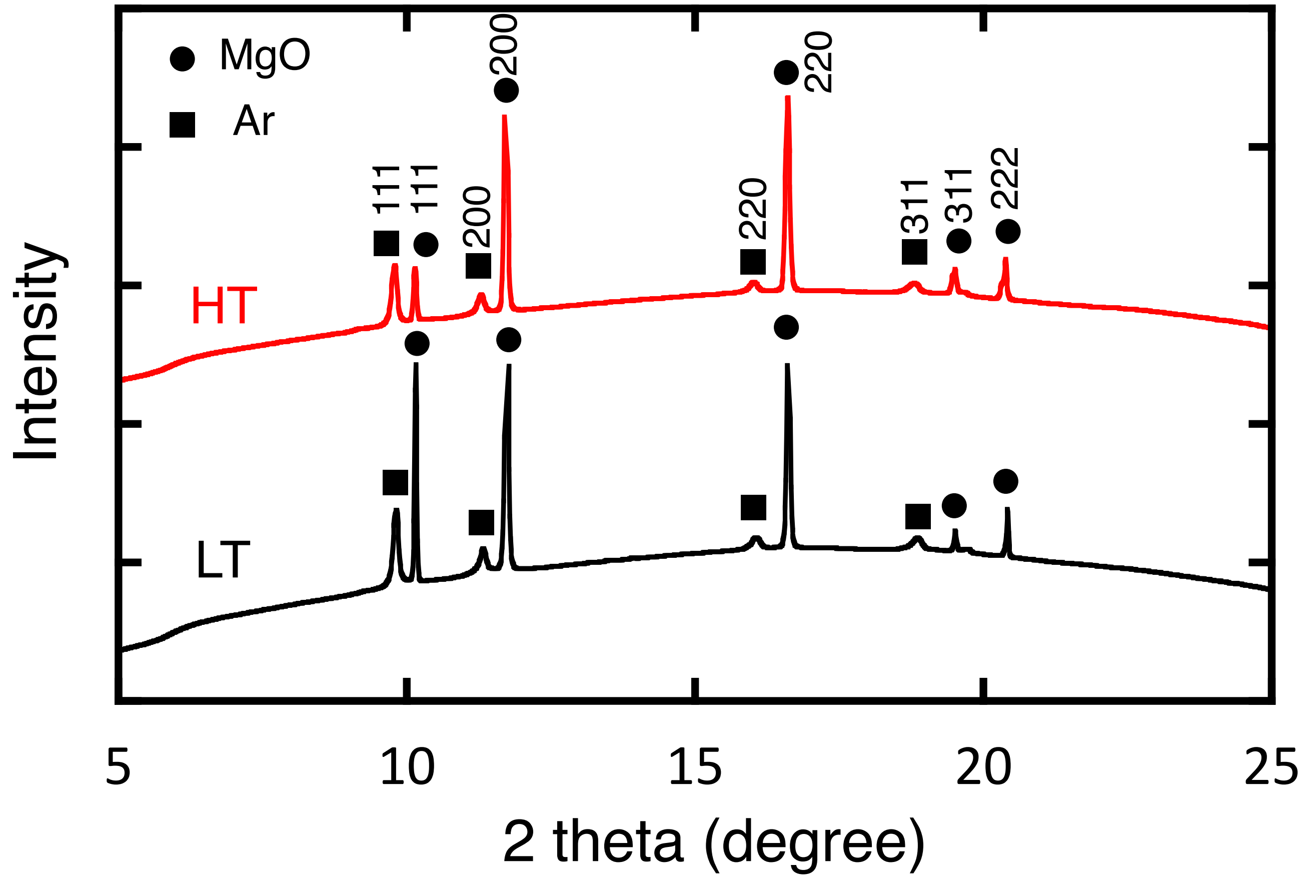

Supplementary Figure 5. XRD patterns of MgO after laser-heating at 5200 K at 23 GPa. The diffraction patterns were collected from the high temperature (HT) (red) and low temperature (LT) (black) regions.

Supplementary Tables

Supplementary Table 1. Observed melting temperature (T_m) of MgO at each pressure condition together with estimated thermal pressure (P_{th}). Errors in the T_m are shown in parentheses.

P (GPa)	P_{th} (GPa)	T_m (K)
8.7	6.6	4150 (200)
14.2	7.3	4550 (220)
17.7	7.9	4900 (290)
23.1	8.4	5210 (150)
23.4	8.1	5050 (70)
30.5	8.5	5250 (300)
36.3	9.0	5600 (190)

Supplementary Table 2. Chemical compositions of MgO sample quenched from 5200 K at 33 GPa. EDS spectrum was taken from 20 × 20 μm^2 squared area in the laser-heated hot spot. Quantification result was shown in weight percent, normalized to 100 (wt.%) total.

Mg	O	Ar	Total
39.53	60.07	0.39	100

Supplementary Note 1: Chemical analysis of the recovered samples

In laser-heated diamond anvil cell experiments, the reaction between a sample and pressure medium or diamond anvil during heating is sometimes of concern [2]. Therefore, we carefully checked any undesirable chemical reaction using synchrotron X-ray diffraction (XRD) at BL10XU of Spring-8. A monochromatic X-ray beam with a wavelength of 0.4134 Å and a beam size of ca. 15 μm was used. The XRD patterns were collected on a CCD detector with a typical exposure time of 180 sec. XRD patterns obtained from both the high and low temperature regions of the sample after heating up to 5200 K at 23 GPa were perfectly indexed with MgO and no residual peaks were observed (Supplementary Fig. 5). Moreover, STEM-EDS analysis was performed to collect the X-ray maps of Mg, O, Ar, C, and Re from the laser-heated area of the sample recovered from 32 GPa and 5200 K (Supplementary Fig. 3), in addition to the quantification analysis under FE-SEM equipped with an energy-dispersive X-ray spectrometer (EDS; Oxford, X-Max 20) (Supplementary Table 2). We found no evidence of chemical reaction, although a trace amount of Ar, which is derived from Ar inclusions, was detected. We also checked the chemical impurity of the sample quenched from ~3100 K at ambient pressure in the air by micro-Raman spectroscopy and SEM-EDS and detected no signs of secondary phases such as brucite $Mg(OH)_2$.

Supplementary Note 2: Melting of $(Mg,Fe)SiO_3$ perovskite

An earlier laser-heated DAC study [3] determined the melting temperatures of $(Mg,Fe)SiO_3$ perovskite up to ~60 GPa based on the discontinuous change in the laser-power vs temperature profile obtained during heating using a single-sided CO_2 laser heating system. The method involved was probably the same as that used in their latter melting experiments for MgO [4]. Despite that the $(Mg,Fe)SiO_3$ sample was heated to ~5000 K at 63 GPa, neither a significant deformation of the sample nor that of gasket, such as observed in the present study, can be seen in their figure (Fig. 2 of [3]). One possible reason for this is that they used a lower-density phase (enstatite) as the starting material, which

transformed to perovskite during heating accompanied by a large volume reduction resulting in the relaxation of the thermal stress. According to the equation of states of the both phases [5], the volume reduction involved for enstatite-perovskite transition is as high as 10% at 25 GPa. The distinct fractures observed inside the $(Mg,Fe)SiO_3$ sample after heating (Fig. 2B of [3]) may indicate such a stress relaxation during heating.

Supplementary References

[1] Fiquet, G. Andrault, D. Itie, J. P. Gillet, P. and Richet, P. X-ray diffraction of periclase in a laser-heated diamond-anvil cell. *Phys. Earth Planet. Inter.* **95**, 1-17 (1996).

[2] Dewaele, A. Mezouar, M. Guignot, N. and Loubeyre, P. High melting points of tantalum in a laser-heated diamond anvil cell. *Phys. Rev. Lett.* **104**, 255701 (2010).

[3] Zerr, A. and Boehler, R. Melting of (Mg,Fe)SiO_3-perovskite to 625 kilobars: Indication of a high melting temperature in the lower mantle. *Science* **262**, 553-555 (1993).

[4] Zerr, A. and Boehler, R. Constraints on the melting temperature of the lower mantle from high-pressure experiments on MgO and magnesiowüstite. *Nature* **371**, 506-508 (1994).

[5] Stixrude, L. and Lithgow-Bertelloni, C. Thermodynamics of mantle minerals-I. Physical properties. Geophys. J. Int. **162**, 610-632 (2005).
